# Not All Ascending Weakness and Numbness Is Guillain-Barré Syndrome: A Report of Two Cases of Copper Deficiency Myelopathy

**DOI:** 10.7759/cureus.100286

**Published:** 2025-12-28

**Authors:** Natalie L Albright, Danielle Pitter, Karima Benameur

**Affiliations:** 1 Neurology, Emory University School of Medicine, Atlanta, USA

**Keywords:** case report, copper deficiency, copper deficiency myelopathy, guillain-barre syndrome (gbs), myelopathy, neuropathy

## Abstract

Copper deficiency can lead to progressive neurological dysfunction, but its symptoms may overlap with other myeloneuropathies, making diagnosis challenging. Clinical features are often nonspecific presentations that may result in initial consideration of alternative neurologic conditions. We present two cases in which copper deficiency was identified after an evolving clinical picture. The first is a 70-year-old woman with a history of esophageal and breast cancer, complicated by esophagectomy and jejunostomy tube feeding, who developed six months of progressive ascending sensory loss and gait instability. The second is an 85-year-old woman with chronic zinc supplementation and denture adhesive use, presenting with two weeks of ascending paresthesias and imbalance. These cases highlight the importance of considering nutritional and surgical history in the evaluation of progressive myeloneuropathy, as well as key physical examination, laboratory, and imaging findings. Clinicians should maintain a high index of suspicion for copper deficiency in patients presenting with Guillain-Barré syndrome (GBS)-like symptoms, particularly those with risk factors for malabsorption or chronic zinc exposure.

## Introduction

Progressive ascending weakness and sensory loss are hallmark features of a wide range of neurological disorders, encompassing both peripheral and central nervous system etiologies ​[1]. While Guillain-Barré syndrome (GBS), an acute inflammatory demyelinating polyneuropathy, is often the first diagnosis considered in patients with progressive, ascending symmetric sensorimotor symptoms ​[2], clinicians must also recognize that nutritional and metabolic myeloneuropathies can present with overlapping features, particularly when both upper and lower motor neuron signs are subtle or evolving. 

Central nervous system disorders, such as copper deficiency myelopathy (CDM), are one such condition, a rare but increasingly recognized cause of progressive sensory ataxia, paresthesias, and gait disturbance ​[3]. Copper plays a critical role in myelin synthesis, mitochondrial function, and hematopoiesis ​[4]. Its deficiency leads to dorsal column dysfunction and macrocytic anemia, classically mimicking subacute combined degeneration ​[3]. The most common etiologies include malabsorption following gastrointestinal surgery, excess zinc intake, and prolonged enteral feeding that bypasses the stomach and duodenum, the primary sites of copper absorption ​[3]. 

Although uncommon, the clinical impact of CDM is significant because neurological deficits may become irreversible without timely recognition and repletion ​[3]. CDM remains underdiagnosed due to its insidious onset, the overlap with more common neuropathies, and the omission of serum copper levels from standard neuropathy workups. 

Here, we present two cases of CDM, initially mistaken for GBS, with distinct etiologic mechanisms: one secondary to enteral feeding via jejunostomy and another due to chronic zinc supplementation. These cases highlight the diagnostic complexity of nutritional myeloneuropathies and underscore the importance of including copper deficiency in the differential diagnosis of progressive sensory and gait disturbances, particularly in patients with gastrointestinal surgery or zinc exposure. These represent two consecutive cases of CDM evaluated at our institution.

## Case presentation

Case 1

A 70-year-old female patient with a history of pulmonary embolism and esophageal and breast cancer status post-chemotherapy, radiation, and esophagectomy 15 years prior, was found down and to be in cardiac arrest in October of 2024. Her husband initiated CPR, after which EMS arrived and restored a pulse, and she was admitted to an outside hospital intensive care unit (ICU). Her post-esophagectomy course had been complicated by a gastric hernia, staple line leak, and gastric conduit fistula into the right lower lobe, necessitating jejunostomy tube (J-tube) placement. She had suffered multiple episodes of pneumonia and required repeated surgical interventions. CT imaging performed at the outside hospital revealed a large right hemopneumothorax with leftward mediastinal shift. She was transferred to our institution, where she was known to the thoracic surgery service, for potential surgical intervention. 

Once the patient was alert, she reported that six months before admission, she began experiencing worsening ascending sensory loss and weakness, progressing to significant gait difficulty one month before presentation. She had a longstanding history of chemotherapy-induced neuropathy from treatment 15 years prior. The neurology service was consulted for concern of GBS dysautonomia causing the cardiac arrest. 

On neurological examination, the patient had normal sensorium and cranial nerves, normal muscle strength, but absent proprioception and sensation of vibration up to the knees, along with hyperreflexia in the lower extremities. The differential diagnosis at this point included an upper motor neuron lesion, given the presence of hyperreflexia, with lower suspicion for GBS, as patients with GBS typically exhibit hypo- or areflexia. 

**Figure 1:**
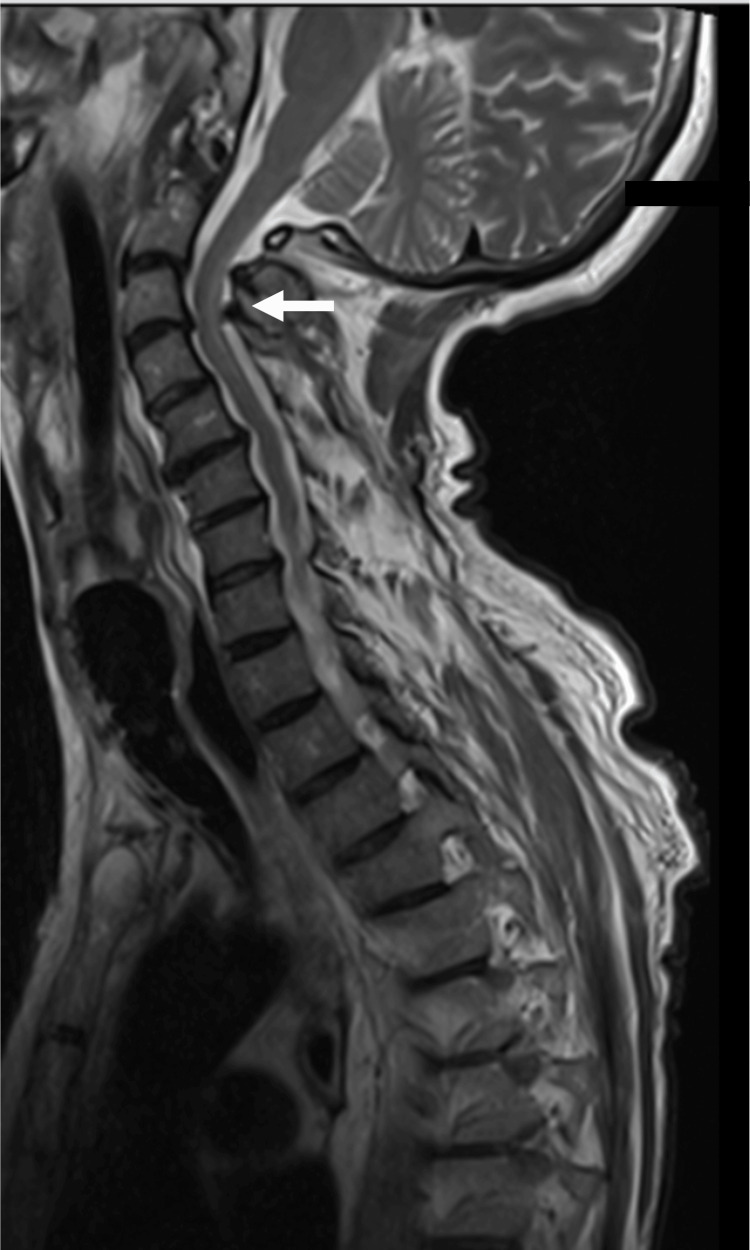
Sagittal T2 MRI of the cervical spine showing T2 hyperintensity in the dorsal columns

MRI of the brain demonstrated incidental punctate acute infarcts in bilateral anterior and posterior circulation territories, likely secondary to cardioemboli from the cardiac arrest; MRI of the cervical spine revealed nonenhancing T2 hyperintensities predominantly involving the dorsal columns (Figure 1), raising suspicion for nutritional deficiency-related myelopathy, such as subacute combined degeneration. Relevant laboratory findings are detailed in Table 1. Workup revealed a macrocytic anemia with stomatocytes, low serum copper, low-normal ceruloplasmin, and mildly elevated serum B12. CSF analysis showed no pleocytosis or albuminocytologic dissociation, as well as normal protein, with negative cytology for malignant cells. Additional testing demonstrated normal levels of vitamin E, vitamin B1, and folate. Human immunodeficiency virus (HIV), rapid plasma reagin (RPR), serum myelin oligodendrocyte glycoprotein immunoglobulin (GMOG-IgG), and both serum and CSF neuromyelitis optica (NMO)-IgG antibodies were negative. Electromyography/nerve conduction study (EMG/NCS) was not obtained due to its nonavailability in the intensive care unit. 

**Table 1:** Relevant laboratory findings in case 1 MCV: mean corpuscular volume

Test	Result	Reference range
MCV	113.2 fL	80-100 fL
Serum copper	35 µg/dL	80-155 µg/dL
Ceruloplasmin	20 mg/dL	20-60 mg/dL
Vitamin B12	1386 pg/mL	180-914 pg/mL
Homocysteine	3 µmol/L	0-15 µmol/L
Methylmalonic acid	0.12 µmol/L	0-0.4 µmol/L

The patient’s history of J-tube feeding, placed distal to the primary sites of copper absorption, the stomach and duodenum, as well as her chronic tube feeds, raised concern for insufficient copper intake or absorption. Her enteral formula included Kate Farms peptide 1.5 65 ml x 12 hours during the day and 45 ml/hour x 12 hours overnight via percutaneous endoscopic gastrostomy (PEG) (4 cartons daily). This included copper of 0.5 mg per carton and zinc of 6 mg per carton ​[5]. Though she had zinc dental fillings, serum zinc levels were within normal limits, making zinc-induced copper deficiency less likely. 

One week after admission, the patient was successfully extubated. She was started on 2.4 mg IV copper repletion for six days and then switched to 2 mg PO daily. She was transferred from the ICU to the medical floor two weeks later but required readmission to the ICU the following day for altered mental status and hypercapnic respiratory failure, necessitating bilevel positive airway pressure (BiPAP) support. Despite intermittent improvements, her condition deteriorated, with increasing oxygen requirements, persistent lethargy, worsening renal function, and persistent pleural effusions. There were no neurological improvements before her decline. 

Given her progressive decline and poor prognosis, palliative care was consulted approximately 2.5 weeks into her hospitalization. After discussions with her family, the decision was made to transition to comfort-focused care. The patient passed away peacefully 3.5 weeks after her arrival at our institution.

Case 2

An 85-year-old female patient with a history of 50 mg zinc supplementation since the COVID-19 pandemic, Fixodent use for 25 years, hypertension, multilevel spinal stenosis, and esophageal strictures, presented with worsening ascending paresthesias and imbalance for two weeks in November of 2024. The neurology service was consulted for possible GBS. Neurologic examination revealed intact cranial nerves, 5/5 strength throughout all extremities, 2+ reflexes in upper extremities, 2+ knee jerk bilaterally, absent ankle reflexes bilaterally, diminished vibration sensation up to the knees, temperature up to the mid-shins, and proprioception in toes bilaterally consistent with a sensory predominant polyneuropathy or myeloneuropathy, given the preserved reflexes. 

Relevant laboratory findings are detailed in Table 2. Additional laboratory studies, such as RPR, thyroid-stimulating hormone (TSH), homocysteine, methylmalonic acid, hemoglobin A1c, serum NMO/AQ4, autoimmune, and heavy metal workup, were unremarkable. Spinal fluid could not be collected as a fluoroscopy-guided lumbar puncture was unsuccessful due to her severe scoliosis. 

**Table 2:** Relevant laboratory findings in case 2

Test	Result	Reference range
MCV	104.3 fL	80-100 fL
Serum copper	<10 µg/dL	80-155 µg/dL
Ceruloplasmin	13 mg/dL	20-60 mg/dL
Vitamin B1	56 nmol/L	70-180 nmol/L
Vitamin B6	10.7 nmol/L	20-125 nmol/L
Vitamin B12	224 pg/mL	180-914 pg/mL
Serum zinc	120.9 µg/dL	60-120 µg/dL

**Figure 2:**
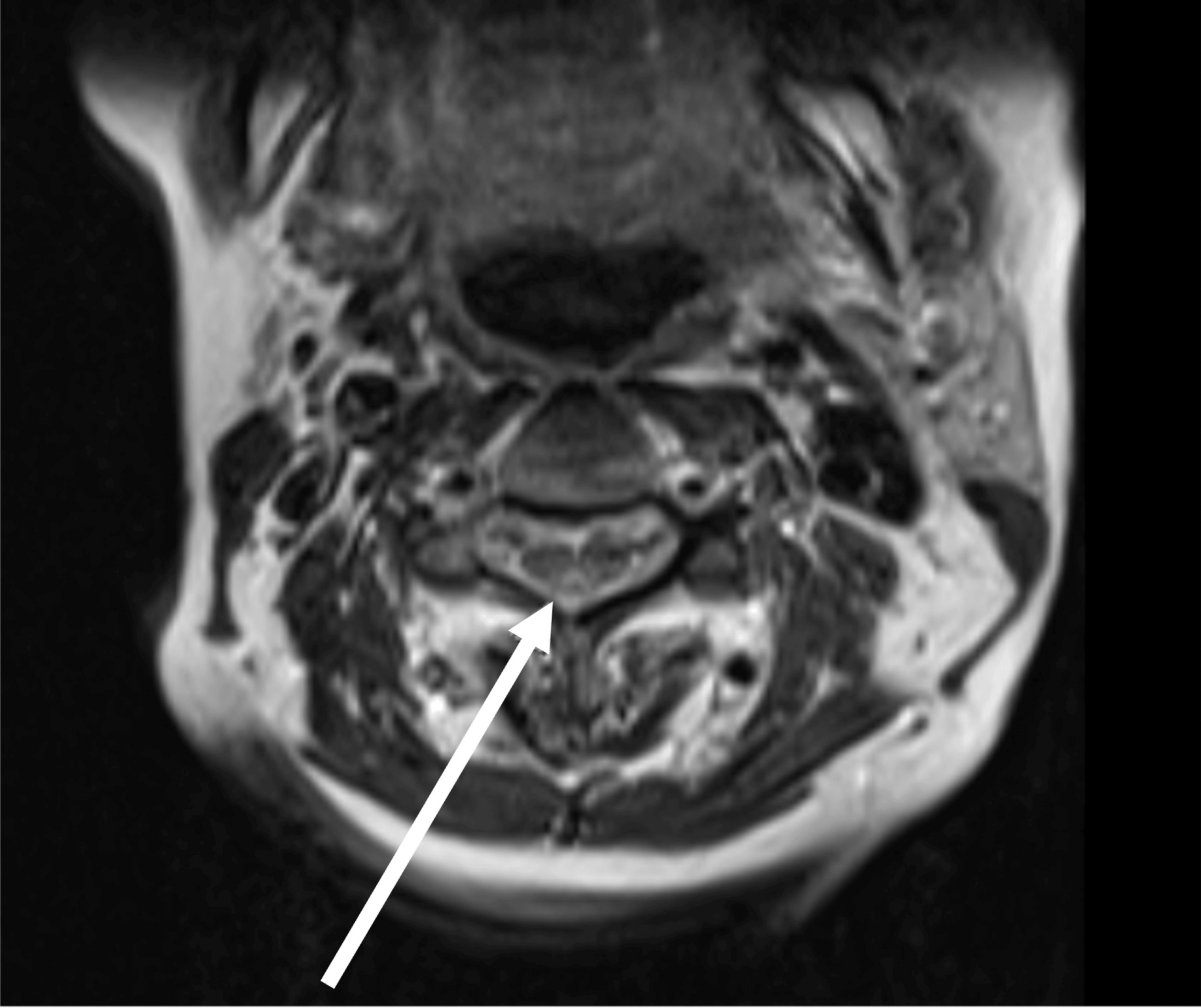
Axial T2 hyperintensity in the dorsal columns spanning C4-C5

CT head was unremarkable. MRI of the C-, T-, and L- spine showed T2-weighted signal abnormality in the dorsal cord at C4 and C5 (Figure 2) and enhancement of the left-sided cauda equina roots. 

EMG was offered, but the patient refused this diagnostic study. Ultimately, she was treated with 2.4 mg cupric chloride daily for five days, then 2 mg copper gluconate. Other vitamin deficiencies were repleted, and she was advised to discontinue her zinc supplementation. Upon vitamin repletion, the patient’s sensory exam began to improve, specifically in regard to temperature. Unfortunately, after discharge, the patient was lost to follow-up. 

## Discussion

CDM is a rare but important cause of progressive myelopathy resulting from inadequate copper intake or impaired absorption [3]. Copper is essential to myelin synthesis, hematopoiesis, and mitochondrial function ​[4,6]. It is primarily absorbed in the stomach and duodenum, and its uptake can be significantly reduced by excess zinc, which induces metallothionein in enterocytes, binding copper and preventing its systemic absorption ​[3,7]. Copper is then transported throughout the body via ceruloplasmin, a copper-carrying protein produced in the liver [4]. 

Risk factors for copper deficiency include gastrointestinal surgeries (e.g., gastric bypass, esophagectomy) ​[8], chronic zinc supplementation ​[9,10], and malabsorptive conditions (such as celiac disease or inflammatory bowel disease) ​[3]. In our first patient, copper malabsorption was likely due to jejunostomy tube placement distal to the primary absorption sites, as the main absorption points for copper are the stomach and proximal small intestine, including the duodenum and proximal jejunum [3]. Thus, distal jejunal feeding without copper repletion can precipitate hypocupremia. CDM occurs in 10% of patients following gastric bypass ​[8]. Despite this, CDM may be underrecognized as its clinical presentation can resemble more common neurological conditions, such as GBS or vitamin B12 deficiency, and clinicians may overlook copper levels in standard neuropathy workups ​[11]. 

In our second patient, excessive zinc intake was the most likely contributor to the development of CDM. Concurrent deficiencies in thiamine (B1), pyridoxine (B6), and borderline cobalamin (B12) may have contributed to the patient’s neuropathic symptoms and macrocytosis. These were corrected during treatment, but the relative contribution of each deficiency cannot be excluded. Although zinc-induced copper deficiency is well-recognized ​[12], to our knowledge, there are no studies demonstrating that excessive zinc intake directly causes depletion of thiamine (B1) or pyridoxine (B6). Experimental data suggest interactions between zinc and B-vitamin status, but the directionality and clinical significance of these relationships remain unclear ​[13]. The observed B-vitamin deficiencies in this patient are therefore more likely attributed to inadequate intake, malabsorption, medication effects, or underlying comorbidities rather than zinc excess itself. 

Clinically, CDM typically presents with sensory ataxia, paresthesias, macrocytic anemia, and characteristic dorsal column signal changes on spinal MRI ​[3,6,14]. One of the most common misdiagnoses is subacute combined degeneration from vitamin B12 deficiency, given the overlap in clinical and radiologic features ​[14]. However, a key distinguishing factor is the presence of normal B12 levels alongside low copper levels. 

Several prior reports have described CDM in settings that overlap with our patients’ risk factors. Kumar et al. presented a series of patients who developed CDM after gastric bypass surgery, highlighting sensory ataxia, macrocytic anemia, and dorsal column signal abnormalities on MRI as key diagnostic clues ​[11]. This seminal report established bariatric surgery as a major risk factor for copper deficiency due to bypassing the primary sites of copper absorption. Patients have also developed CDM secondary to chronic excessive zinc exposure from denture creams [10]. This demonstrated the critical mechanism by which zinc induces metallothionein production in enterocytes, binding copper and causing systemic copper deficiency. These cases underscore the need for heightened clinical suspicion for CDM in patients with risk factors such as gastrointestinal surgery or excessive zinc exposure. 

Our case series underscores the importance of maintaining a broad differential diagnosis when evaluating ascending sensorimotor symptoms. Our first case is unique in the concurrent presentation of chemotherapy-induced neuropathy and copper deficiency, which, to our knowledge, has not been reported in the literature. This highlights the diagnostic challenge when multiple etiologies contribute to neurological deterioration, and the importance of considering central nervous system pathology when hyperreflexia and dorsal column signs are present. Subtle examination findings such as hyperreflexia should raise suspicion for central nervous system involvement rather than peripheral neuropathy like GBS ​[3]. While both GBS and CDM can present with ascending sensory loss, GBS typically demonstrates hypo- or areflexia [15], whereas CDM is associated with hyperreflexia due to dorsal column dysfunction ​[3]. Additionally, macrocytic anemia, a hallmark of CDM, is not a feature of GBS and can serve as an important diagnostic clue ​[6]. 

MRI findings can further aid differentiation. Dorsal column T2 hyperintensities are characteristic of CDM and other central myelopathies but are not seen in GBS ​[14], which primarily affects peripheral nerves and nerve roots. In our second patient, there is unclear significance of the root enhancement as we could not obtain CSF studies; however, the patient did not have any radicular symptoms. 

Treatment of copper deficiency involves prompt copper supplementation, which can be administered intravenously or orally depending on severity and the patient’s ability to absorb enteral copper. Published series have successfully used oral copper supplementation of 2-4 mg/day, sometimes preceded by a short course of parenteral therapy, with escalation up to 8 mg/day in cases of relapse or persistent deficiency ​[3]. Treatment response can be monitored with serial serum copper and ceruloplasmin levels, as well as tracking hematologic indices (e.g., mean corpuscular volume) and neurological exam findings for signs of stabilization or improvement ​[12]. In patients at high risk for developing CDM, it is recommended that copper levels be routinely monitored after gastrointestinal surgeries or by adding copper to tube feeds ​[8]. While hematologic response is often prompt within weeks of initiating supplementation, neurological prognosis remains guarded; a systematic review found that only 24% of patients experienced clinical improvement and just 5.1% recovered fully ​[12,16]. The symptoms that are most likely to persist are sensory deficits, especially proprioceptive loss and sensory ataxia, and gait instability ​[9,12]. These outcomes highlight the importance of early recognition and intervention before irreversible damage occurs. 

Limitations of our report include incomplete electrophysiologic data and a lack of follow-up imaging, which limit definitive attribution of all neurological findings to copper deficiency alone. 

Ultimately, in patients presenting with ascending weakness or numbness, hyperreflexia, and macrocytic anemia, particularly those with risk factors such as gastrointestinal surgery or chronic zinc use, clinicians should consider CDM in the differential diagnosis. Thorough history-taking regarding surgeries and supplement use, plus integration of systemic and neurologic findings, is essential to avoid premature closure on diagnoses like GBS. Early diagnosis and treatment are key to preventing permanent neurological deficits.

## Conclusions

CDM is a rare but important cause of progressive myelopathy, often overlooked due to its clinical resemblance to more commonly known neurological disorders, such as subacute combined degeneration, given the similar imaging. Both of our patients presented with ascending weakness and numbness, leading the primary teams to consult neurology for possible GBS. Our cases highlight the critical importance of considering CDM in patients presenting with ascending sensory symptoms, hyperreflexia, and macrocytic anemia. Early recognition, thorough history-taking, and prompt copper supplementation are essential to prevent irreversible neurological deficits. Clinicians should maintain a high suspicion for CDM in patients showing central neurological examination findings suggestive of myelopathy rather than neuropathy to ensure timely intervention and better patient outcomes. 
